# Two Novel *CYP11B1* Gene Mutations in Patients from Two Croatian Families with 11**β**-Hydroxylase Deficiency

**DOI:** 10.1155/2014/185974

**Published:** 2014-06-02

**Authors:** Katja Dumic, Tony Yuen, Zorana Grubic, Vesna Kusec, Ingeborg Barisic, Maria I. New

**Affiliations:** ^1^Division of Clinical Genetics, Department of Pediatrics, Children's University Hospital Zagreb, Klaiceva 16, 10000 Zagreb, Croatia; ^2^Steroid Disorder Program, Department of Pediatrics, Mount Sinai School of Medicine, New York, NY 10029, USA; ^3^Tissue Typing Centre, University Hospital Centre Zagreb, 10000 Zagreb, Croatia; ^4^Department of Laboratory Medicine, University Hospital Centre Zagreb, 10000 Zagreb, Croatia

## Abstract

Steroid 11**β**-hydroxylase deficiency (11**β**-OHD) is the second most common cause of congenital adrenal hyperplasia. Mutations in the *CYP11B1* gene, which encodes steroid 11**β**-hydroxylase, are responsible for this autosomal recessive disorder. Here, we describe the molecular genetics of two previously reported male siblings in whom diagnosis of 11**β**-OHD has been established based on their hormonal profiles displaying high levels of 11-deoxycortisol and hyperandrogenism. Both patients are compound heterozygous for a novel p.E67fs (c.199delG) mutation in exon 1 and a p.R448H (c.1343G>A) mutation in exon 8. We also report the biochemical and molecular genetics data of one new 11**β**-OHD patient. Sequencing of the *CYP11B1* gene reveals that this patient is compound heterozygous for a novel, previously undescribed p.R141Q (c.422G>A) mutation in exon 3 and a p.T318R (c.953C>G) mutation in exon 5. All three patients are of Croatian (Slavic) origin and there is no self-reported consanguinity in these two families. Results of our investigation confirm that most of the *CYP11B1* mutations are private. In order to elucidate the molecular basis for 11**β**-OHD in the Croatian/Slavic population, it is imperative to perform *CYP11B1* genetic analysis in more patients from this region, since so far only four patients from three unrelated Croatian families have been analyzed.

## 1. Introduction

Congenital adrenal hyperplasia (CAH) is a group of autosomal recessive disorders caused by the loss of one of five steroidogenic enzymes involved in cortisol synthesis. Approximately 90–95% of all cases are due to steroid 21-hydroxylase deficiency, and about 3–8% are caused by steroid 11*β*-hydroxylase deficiency (11*β*-OHD) [1–3]. The deficiency of 11*β*-OH leads to reduced cortisol biosynthesis, increased ACTH secretion, and overproduction of steroid precursors. These precursors are shunted toward androgen synthesis, resulting in hyperandrogenism. Phenotypical expression of classic 11*β*-OHD leads to virilization of external genitalia in newborn females. The overproduction of reactive androgen also causes precocious pseudopuberty, accelerated somatic growth, and premature epiphyseal closure in both sexes. The accumulation of 11-deoxycorticosterone and its metabolites causes hypertension in about two-thirds of these patients.

The gene for steroid 11*β*-hydroxylase is encoded by* CYP11B1*. It is located on chromosome 8q22, approximately 40 kb apart from the highly homologous* CYP11B2* gene that encodes the aldosterone synthase. To date, over 90 disease-causing* CYP11B1* mutations have been identified [1, 4, 5]. Notably, a high incidence of 11*β*-OHD has been reported, with a disease frequency of about 1 in 5,000–7,000 live births, in Israel among Jewish immigrants from Morocco, a relatively inbred population. Almost all alleles in this patient group carry the same p.R448H (c.1343G>A, rs28934586) missense mutation in exon 8 [6–8]. Recently, we described one 11*β*-OHD patient of the Slavic origin who was compound heterozygous for this p.R448H mutation and a novel intron 7 (c.1200+4A>G) splice site mutation [9]. It was the first report of* CYP11B1* genetic analysis in a Croatian patient with 11*β*-OHD.

Here, we further report two novel* CYP11B1* mutations from three more patients of the Croatian descent, which include two previously reported brothers [10] and one new patient.

## 2. Subjects and Methods

### 2.1. Subjects

#### 2.1.1. Family A

Patient 1 is the older of two sons of healthy nonconsanguineous parents of the Croatian descent. He was diagnosed at 2.5 years of age due to accelerated growth, skeletal maturation, pseudoprecocious puberty, elevated serum levels of 11-deoxycortisol, 17-hydroxyprogesterone (17-OHP), and androgens, and suppressed levels of cortisol, aldosterone, and plasma renin activity (PRA). His blood pressure was high normal. Patient 2 is the younger brother of Patient 1. He was diagnosed at 3 months of age, with clinical presentation almost identical to his older brother, except for a normal blood pressure. Hydrocortisone treatments for both patients were introduced immediately after diagnosis.

Patients 1 and 2, now 30 and 28 years old, respectively, have not been under our pediatric care or taking medication on a regular basis for the past 15 years. They came to us recently for genetic counseling. During this visit, blood was drawn for* CYP11B1* gene analysis.

#### 2.1.2. Family B

Patient 3, the first child of healthy nonconsanguineous parents of the Croatian descent, was born spontaneously at term after an uneventful pregnancy. At 2 years of age, he was presented for the first time with growth acceleration and pseudoprecocious puberty. His height was 100 cm (+4.27 SD) and his weight was 19.5 kg (+3.9 SD). He had deep voice, acne, and large phallus (length 7 cm, circumference 4.5 cm). His testes were 2-3 cc, and his pubic hair was Tanner II. Blood pressure was normal (90/45 mmHg). Bone age according to Greulich and Pyle was 5 years. Plasma electrolytes were normal. Biochemical results confirmed diagnosis of 11*β*-OHD (Table 1) and hydrocortisone treatment (12 mg/m^2^/day) was subsequently started.

His younger brother was admitted at 6 months of age. He had no signs of accelerated growth and sexual development, and his plasma levels of 11-deoxycortisol, 17-OHP, androgens, aldosterone, and PRA were within normal range for age, suggesting that he was not affected with CAH.

### 2.2. Hormonal Assays

Blood for biochemical analyses was drawn after an overnight fast. Serum/plasma was either assayed immediately or frozen for later use. Standard recommended biochemical methods for measured parameters were employed.

### 2.3. DNA Amplification and Sequence Analysis

Genomic DNA was isolated from peripheral leukocytes. Three DNA fragments (exons 1-2, 3–5, and 6–9) of the* CYP11B1* gene were amplified by polymerase chain reaction (PCR). Reactions were carried out in 50 *μ*L volume, which contained 100 ng genomic DNA, 10 mM Tris-HCl, pH 8.3, 50 mM potassium chloride, 1.5 mM magnesium chloride, 200 *μ*M deoxynucleotide triphosphates, 2.5 units JumpStart* Taq* DNA Polymerase (Sigma, St. Louis, MI, USA), and 200 nM each of* CYP11B1*-specific sense and antisense primers (Table 2). Thermocycling was performed in a Mastercycler (Eppendorf, Hauppauge, NY, USA) with an initial 3-minute denaturation step at 94°C followed by 35 cycles of 94°C for 45 seconds, 58°C for 30 seconds, and 72°C for 3 minutes. The expected amplicon sizes were 0.9 kb, 1.4 kb, and 1.5 kb for the exon 1-2, 3–5, and 6–9 fragments, respectively, and were confirmed by agarose gel electrophoresis. After purification with a QIAquick PCR Purification Kit (Qiagen, Valencia, CA, USA), direct sequencing of the amplicons was performed using primers listed in Table 2. The sequencing results were compared to the 7.46 kb human* CYP11B1* reference sequence at chromosome 8 (GenBank gi|224589820:c143961236-143953773).

## 3. Results

**Figure 1:**
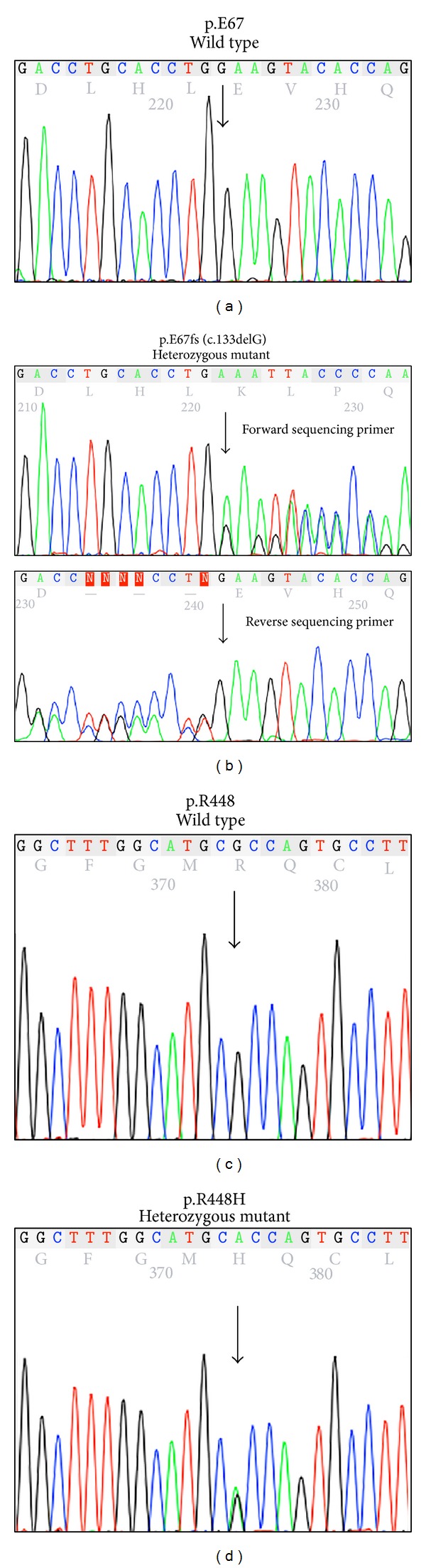
Sequencing electropherograms showing* CYP11B1* mutations in Patient 1. Wild-type* CYP11B1* DNA sequences encoding p.E67 (a) and p.R448 (c) from a normal individual are shown as reference. Patient 1 is compound heterozygous for a novel exon 1 p.E67fs (c.199delG) frameshift mutation on one allele (b) and an exon 8 p.R448H mutation on the other allele (d) of the* CYP11B1* gene. Patient 2, the younger brother of Patient 1, carries identical mutations.

More than two decades ago, at a time before modern molecular diagnostic tools were available, we described two brothers (Patients 1 and 2) with 11*β*-OHD [9]. These two patients recently visited our clinic for genetic counseling. This gave us an opportunity to perform genetic analysis of their* CYP11B1* gene. DNA sequencing results showed that both patients carried a novel p.E67fs (p.E67Kfs∗9, c.199delG) frameshift mutation in exon 1 and a previously reported p.R448H (c.1343G>A) missense mutation in exon 8 (Figures 1 and 3). The p.E67fs mutation causes a reading frame shift, resulting in premature translation termination at codon 75.

**Figure 2:**
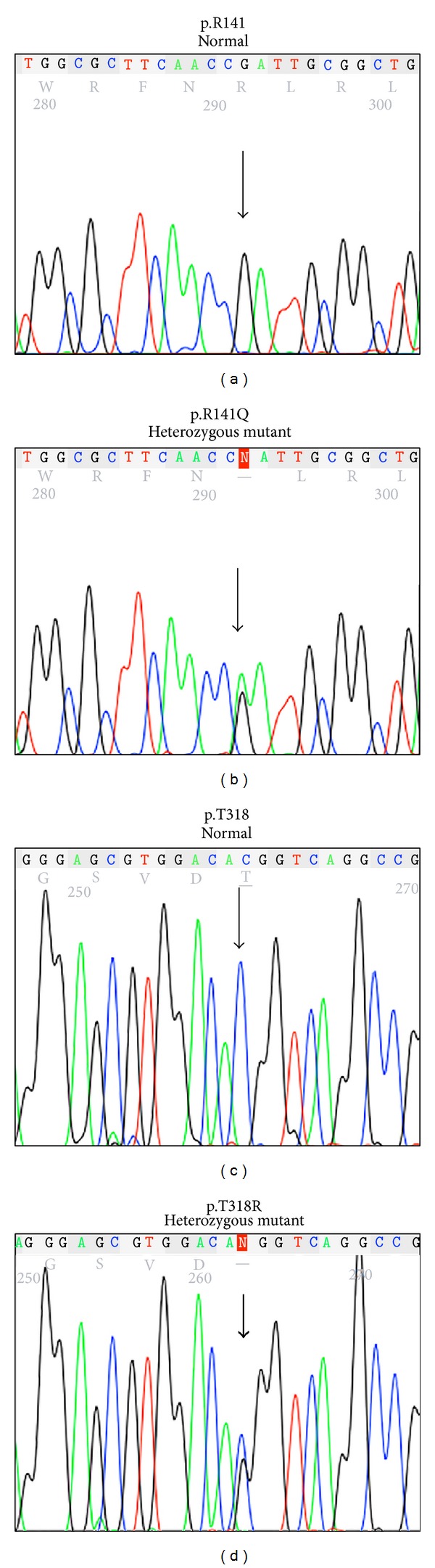
Sequencing electropherograms showing* CYP11B1* mutations in Patient 3. Wild-type* CYP11B1* DNA sequences encoding p.R141 (a) and p.T318 (c) from a normal individual are shown as reference. Patient 3 is compound heterozygous for a novel exon 3 p.R141Q mutation on one allele (b) and an exon 5 p.T318R mutation on the other allele (d) of the* CYP11B1* gene.

**Figure 3:**
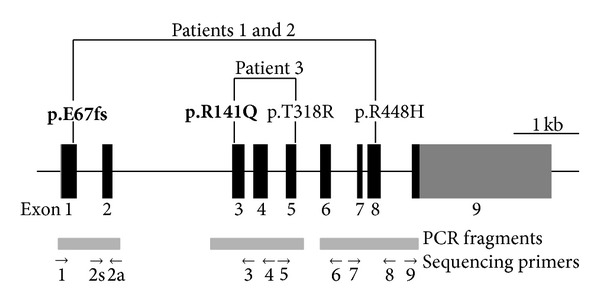
Schematic diagram of the human* CYP11B1* gene showing positions of mutations identified in this study. The two novel mutations, p.E67fs (c.199delG) in exon 1 and p.R141Q (c.422G>A) in exon 3, are indicated in bold type.

For Patient 3, deficiency of 11*β*-OH was suspected on the basis of his clinical presentation. This is confirmed by hormonal analyses, which showed elevated serum levels of 11-deoxycortisol, androstenedione, testosterone, and 17-OHP and suppressed levels of cortisol, aldosterone, and plasma renin activity (Table 1). Genetic analysis of the* CYP11B1* gene revealed that Patient 3 carried a novel, previously undescribed p.R141Q (c.422G>A, rs26701810) missense mutation in exon 3 and a previously reported p.T318R (c.953C>G) missense mutation in exon 5 (Figures 2 and 3) [11].

In both families, DNA of the parents was not available for genetic analysis. Since all three patients have clear clinical and biochemical characteristics of 11*β*-OHD, we assume that the patients are compound heterozygotes, and that the mutations are located on different alleles.

## 4. Discussion

Patients 1, 2, and 3 were presented at the age of 2.5 years, 3 months, and 2 years, respectively, with characteristic features for boys with 11*β*-OHD. Accelerated somatic growth and skeletal maturation and pseudoprecocious puberty were found in all three patients. Although their blood pressure was normal, their hormonal profile was distinctive of 11*β*-OHD, with elevated serum levels of 11-deoxycortisol, 17-OHP, and androgens and suppressed levels of cortisol, aldosterone, and PRA.

At present, over 90 different* CYP11B1* gene mutations have been described. Whereas the majority of these are missense and nonsense mutations, other mutations have also been reported, which include splice site mutations, small and large deletions/insertions, and complex rearrangements. These mutations are distributed over the entire coding region but tend to cluster in exons 2, 6, 7, and 8 [1, 2].

**Figure 4:**
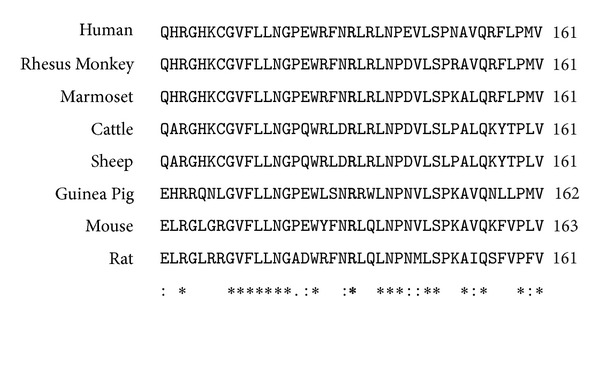
p.R141 is conserved in mammalian* CYP11B1* protein. The GenBank protein sequences used for the alignment were NP_000488.3 (*Homo sapiens*, Human), NP_001180667.1 (*Macaca mulatta*, Rhesus Monkey), XP_002759236.1 (*Callithrix jacchus*, White-Tufted-Ear Marmoset), NP_777063.2 (*Bos taurus*, Cattle), NP_001068568.1 (*Ovis aries*, Sheep), NP_001166410.1 (*Cavia porcellus*, Guinea Pig), NP_001028401.2 (*Mus musculus*, Mouse), and NP_036669.3 (*Rattus norvegicus*, Rat). p.R141 is in bold letter. Fully (asterisk), strongly (colon), and weakly (period) conserved residues are indicated.

There is no consistent correlation between a specific* CYP11B1* gene mutation and the clinical phenotype of 11*β*-OHD. Distinctive phenotypic variability exists in patients with the same mutation regarding the onset of symptoms, the age of diagnosis, the degree of virilization, or the severity of hypertension [1, 2]. Nonetheless, based on* in vitro* expression data, the p.R448H mutation completely abolished 11*β*-hydroxylase enzymatic activity [12]. The c.199delG frameshift mutation should result in premature translational termination and production of a nonfunctional protein. The compound heterozygous c.199delG/p.R448H mutation therefore predicts a severe classic CAH phenotype in Patients 1 and 2. Similarly, the T318 residue is completely conserved in all known P450 enzymes. Any changes in this position, for example, either a p.T318 M or a p.T318R mutation, causes loss of 11*β*-hydroxylase activity [1]. Although the consequence of the p.R141Q mutation is unknown, based on the severe clinical phenotype observed in Patient 3, we predict that the p.R141Q mutation causes major loss of 11*β*-hydroxylase activity. The mutation of a positively charged arginine to an uncharged glutamine residue could break a salt bridge necessary for maintaining conformational stability of the enzyme.* In silico* mutation analyses were performed using PolyPhen-2 (http://genetics.bwh.harvard.edu/pph2/), SIFT (http://sift.jcvi.org/), and Provean (http://provean.jcvi.org/) software. All three software predicted a damaging effect of the p.R141Q mutation on CYP11B1 function (Table 3). That p.R141 is conserved in the mammalian CYP11B1 protein further suggests an important role of this residue at this position (Figure 4).

Most of the reported mutations are private, family-specific mutations. However, in some ethnic groups, particularly in families with high rate of consanguinity, ethnic-specific* CYP11B1* mutations in homozygous forms have been described. These include the p.R448H mutation identified among Jewish immigrants from Morocco [5, 6, 13, 14]. The p.Q356X (c.1066C>T, rs146124466) and p.G379V (c.1136G>T) mutations are associated mostly with 11*β*-OHD patients from Tunisia, although some patients with p.Q356X mutation have also been described in patients originating from Africa [12, 15].

Our families with 11*β*-OHD patients are all of the Croatian (Slavic) origin, and there is no self-reported consanguinity. Mutation analysis of the* CYP11B1 *gene revealed that the p.R448H mutation was present not only in one previously reported Croatian patient [9], but also in Patients 1 and 2. Thus, three patients from two Croatian families carry this mutation, which is otherwise rarely reported in Caucasians [16, 17]. Also, similar to this previously reported patient, all three patients in this study carry novel* CYP11B1* mutations in the heterozygous form. This is consistent with former reports that most of the* CYP11B1* mutations are private because they can only be found within the same family [13].

To the best of our knowledge, there are no other patients with 11*β*-OHD in the Slavic population in whom* CYP11B1* gene analysis has been performed. Therefore, it is imperative to perform* CYP11B1* genetic analysis in more patients from this region. Our investigation should benefit from genetic counseling, prenatal and postnatal diagnosis, and treatment of 11*β*-OHD in Croatia.

## Figures and Tables

**Table 1:** Hormonal analyses of Patient 3.

Hormones	Results	Normal range
11-deoxycortisol (nmol/L)	186	0.1–2.0
17-hydroxyprogesterone (nmol/L)	36	1.5–7.0
Androstenedione (nmol/L)	5	0.3–1.5
Testosterone (nmol/L)	2.2	0.1–0.3
Cortisol (nmol/L)	102	140–690
Aldosterone (pmol/L)	160	200–800
PRA (*μ*gL^−1^h^−1^)	1.1	2.0–5.2

**Table 2:** List of primers for PCR amplification and DNA sequencing of the *CYP11B1* gene.

Primer sequence	Location	Orientation	Purpose
TCGAAGGCAAGGCACCAG	Promoter	sense	Exons 1-2 amplification
TGCTCCCAGCTCTCAGCT	Intron 2	antisense	Exons 1-2 amplification and Exon 1 sequencing
AGAAAATCCCTCCCCCCTA	Intron 2	sense	Exons 3–5 amplification
GACACGTGGGCGCCGTGTGA	Intron 5	antisense	Exons 3–5 amplification
TGACCCTGCAGCTGTGTCT	Intron 5	sense	Exons 6–9 amplification
GAGACGTGATTAGTTGATGGC	Exon 9, 3′ UTR	antisense	Exons 6–9 amplification
CACCAGGCAAGATAAAAG	Promoter	sense	Exon 1 sequencing
AGACACTTTGGATTGGGAC	Intron 1	sense	Exon 2 sequencing
AGGATGCACTGCTGAGCAC	Intron 3	antisense	Exon 3 sequencing
GTGGAGAGGGAGAAATTGGG	Intron 4	antisense	Exon 4 sequencing
AGGATGTTTCCCAGCACCAAAG	Intron 4	sense	Exon 5 sequencing
ATTCCAGAGGAAGAAGAGC	Intron 6	antisense	Exon 6 sequencing
CATGGATCTGGGACCTCTG	Intron 6	sense	Exon 7 sequencing
AGGCCAGTCCCACATTGCTC	Intron 8	antisense	Exon 8 sequencing
CCCCCTTCAGCATAATCTC	Intron 8	sense	Exon 9 sequencing

(Footnote) 3′ UTR: 3′ untranslated region.

**Table 3:** *In silico* prediction of the effects of the p.R141Q mutation in *CYP11B1* function.

Prediction software	Score	Prediction of Effects of Mutation
PolyPhen-2 (http://genetics.bwh.harvard.edu/pph2)	1.000	Probably Damaging
SIFT (http://sift.jcvi.org)	0.000	Damaging
Provean (http://provean.jcvi.org)	−3.769	Deleterious
